# Frequency and pattern of migraine among medical and nursing students at Enugu, South East Nigeria

**DOI:** 10.1186/1129-2377-14-S1-P5

**Published:** 2013-02-21

**Authors:** BA Ezeala-Adikai, OS Ekenze, IO Onwuekwe

**Affiliations:** 1 University of Nigeria Teaching Hospital, Enugu, Nigeria

## Introduction

Headache may be the commonest neurological disorder in the community and may impose a substantial burden on sufferers and on society. Although data from the last century revealed that primary headaches were rare among Africans, newer data have shown higher prevalence in the continent. Few studies have addressed the frequency of migraine among students in South East Nigeria

## Objective

To ascertain the Frequency and pattern of migraine among young Nigerians as represented by medical and nursing students in two Teaching Hospitals and two Nursing Schools in Enugu, South East Nigeria.

## Methods

This was a cross-sectional descriptive interview-based study using structured headache questionnaire. Consent was obtained and the results interpreted following the guidelines of the International Headache Society (II).

## Results

The one year frequency of primary headache of any type was 86.3% (85.4% in males and 86.7% in females). The frequency of migraine was 13.1% (males 10.8%, females 14.8%). Frequency of migraine was highest below 20 years (16%) (males -8%, females 18.8%). The peak frequency for males was from the ages of 20 to 26 (15.3%) and below 20 years for females (18.8%). Most, migraine attacks were unilateral (89.8%), moderate/severe (67.8%), pulsating 96.6%) with phono/photophobia (83.1%) and stress as the commonest triggering factor (61%). Migraine attacks were frequent in 66.1% and affected the quality of life of the sufferers in 40.7%.

## Conclusion

There is a high frequency of headache of migraine among medical and nursing students in Enugu. Migraine was 1.4 times commoner in females than in males. Migraine affected the quality of life of more than 40% of its sufferers.

